# Revisiting epidemiology of leishmaniasis in central Asia: lessons learnt

**DOI:** 10.1017/S0031182022001640

**Published:** 2023-02

**Authors:** Vyacheslav Yurchenko, Daniil S. Chistyakov, Lyudmila V. Akhmadishina, Alexander N. Lukashev, Jovana Sádlová, Margarita V. Strelkova

**Affiliations:** 1 Life Science Research Centre, Faculty of Science, University of Ostrava, 71000 Ostrava, Czech Republic; 2 Martsinovsky Institute of Medical Parasitology, Sechenov University, 119435 Moscow, Russia; 3 Faculty of Bioengineering and Bioinformatics, Lomonosov Moscow State University, 119991 Moscow, Russia; 4 Department of Parasitology, Faculty of Science, Charles University, 12844 Prague, Czech Republic

**Keywords:** Animal reservoir, central Asia, coinfection, great gerbils, *Leishmania gerbilli*, *Leishmania major*, *Leishmania turanica*, *Leishmaniavirus*

## Abstract

In this work we reviewed historical and recent data on *Leishmania* spp. infection combining data collected in Turkmenistan, Uzbekistan, Kazakhstan, Kyrgyzstan, Iran, China and Mongolia. We specifically focused on a complex of co-existing species (*Leishmania major*, *Leishmania turanica* and *Leishmania gerbilli*) sharing the same animal reservoirs and vectors. In addition, we analysed the presence of dsRNA viruses in these species and discussed future research directions to identify species-specific traits, which may determine susceptibility of different *Leishmania* spp. to viral infection.

## Graphical abstract

**Figure:**
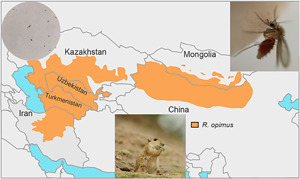

## Introduction

Leishmaniasis is one of the neglected vector-borne tropical diseases endemic in almost 100 countries worldwide caused by *Leishmania* spp. (Euglenozoa: Trypanosomatidae) (Bruschi and Gradoni, 2018; Kostygov *et al*., 2021*b*). Between 10 and 15 million people in the world are infected, and the annual rate of new infections is over 2 million cases (WHO, 2022). The mortality per year from leishmaniasis is second only to malaria among all parasitic diseases (Pace, 2014). Its clinical manifestations range from cutaneous ulcers to systemic multiorgan diseases in the cases of cutaneous leishmaniasis (CL) and visceral leishmaniasis (VL), respectively (Bruschi and Gradoni, 2018).

In the Old World, the main areas of CL circulation are northern Africa, central Asia (hereafter, Kazakhstan, Kyrgyzstan, Mongolia, Turkmenistan and Uzbekistan) and the Middle East (Alvar *et al*., 2012; Torres-Guerrero *et al*., 2017). The most common *Leishmania* spp. documented in the Old World are *Leishmania aethiopica*, *Leishmania major*, *Leishmania tropica* and species of the *Leishmania donovani* complex (*L. donovani* and *Leishmania infantum*) (Lukeš *et al*., 2007; Bruschi and Gradoni, 2018). The VL in humans is mainly caused by members of the *L. donovani* complex and may manifest in damages to the liver, spleen, lymph nodes and bone marrow often resulting in death of a patient, if not diagnosed and treated in a timely manner (Strelkova *et al*., 2015; Mann *et al*., 2021). Infection with these species may also present skin manifestations in the cases of post-kala-azar dermal leishmaniasis or atypical leishmaniases (Guan *et al*., 2013; Zhang *et al*., 2014; Ben-Shimol *et al*., 2016; Zijlstra, 2016). *Leishmania aethiopica*, *L. major* and *L. tropica* mostly cause CL, although some isolates of *L. major* and *L. tropica* were occasionally identified from patients with VL (Alborzi *et al*., 2008; Bruschi and Gradoni, 2018; Charyyeva *et al*., 2021). The CL can be further subdivided into anthroponotic (ACL) and zoonotic (ZCL) forms, which are predominantly caused by *L. tropica* and *L. major*, respectively (Akilov *et al*., 2007; Ghatee *et al*., 2020). Great gerbils (*Rhombomys opimus*) and fat sand rats (*Psammomys obesus*) serve as the main animal reservoirs for ZCL in central Asia and the Middle East, correspondingly (Elfari *et al*., 2005; Akhavan *et al*., 2010*c*), although other animal species – for example, Libyan jird (*Meriones libycus*), Shaw's jird (*Meriones shawi*), Indian gerbil (*Tatera indica*) or Indian desert gerbil (*Meriones hurrianae*) – may play this role in particular geographic regions (Yaghoobi-Ershadi *et al*., 1996; Rassi *et al*., 2001; Mohebali *et al*., 2004; Parvizi *et al*., 2008; Ghawar *et al*., 2011; Akhoundi *et al*., 2013).

The great gerbils may simultaneously host several species of *Leishmania*. In addition to pathogenic to humans *L. major*, they may also be infected by gerbil-restricted *Leishmania turanica* and *Leishmania gerbilli* (Strelkova *et al*., 1990*b*, 2001; Akhavan *et al*., 2010*b*). Here, we reviewed the literature on the mixed infections of *L. major*, *L. turanica* and *L. gerbilli* in central Asia and neighbouring countries with a focus on their natural animal reservoirs. As a second aim, we wanted to highlight some important papers on this topic published in Russian, and, as such, not well-known to the researchers in other countries.

## Historical notes

Parasites of the genus *Leishmania* were first formally described by Leishman and Donovan in 1903 in patients infected with kala-azar in India (Donovan, 1903; Leishman, 1903). The parasite causing tropical ulcer was described as *Helcosoma tropicum* by Wright in the same year (Wright, 1903) and renamed as *Leishmania tropica* in 1906 by Lühe (1906). Yet, the first scientist documented the presence of a parasite now known as *L. tropica* was Cunningham in 1885 (Cunningham, 1885). Its protistan nature (‘class of protozoa’) was discovered by Borovsky (1898), but remained unrecognized until much later (Hoare, 1938). In 1914, Yakimov identified 2 variants of *Leishmania* sp., based on the size of amastigotes in the macrophages of patients and named them *L. tropica minor* and *L. tropica major* (Yakimov and Schokhor, 1914). Later studies revealed that *L. t. minor* causes dry ulcers usually lasting for over a year and it is more commonly spread in the cities. In contrast, *L. t. major* manifests in wet ulcers, the course of the disease is shorter and it is more commonly spread in rural areas (Latyshev and Krukova, 1941; Kozevnikov, 1963; Schnur, 1987). In 1973, summarizing the accumulated data, Bray proposed to reclassify parasites as *L. tropica* for the causative agent of ACL and *L. major* for the causative agent of ZCL (Bray *et al*., 1973).

It is important to note that for about 50 years all isolates of *Leishmania* coming from animals and people in central Asia and neighbouring countries with a characteristic clinical picture were classified as *L. major*, with the only exception being a description of another *Leishmania* sp., *L. gerbilli*, from *R. opimus* in 1964 in China (Wang *et al*., 1964). In line with that, *in vitro* experiments in animals demonstrated that different isolates have different levels of virulence. The highly virulent (HV) strains were invariably isolated from humans. They caused a progressive disease with obligatory ulceration in golden hamsters and domestic mice. Conversely, the strains with virulence ranging from low (low virulent strains, LV) to high could be isolated from gerbils. The LV strains caused a slow course of the disease that was limited to infiltrates and never led to ulceration. The strains with intermediate virulence caused a prolonged disease manifesting in small abortive ulcers in the later stages (Kellina, 1965; Lavrova *et al*., 1973; Kellina *et al*., 1981). Experimental infection of different animal species (in which *Leishmania* presence was documented in nature) with clonal cultures or strains of *Leishmania* with different virulence (HV and LV) revealed that HV parasites infected all the tested animals – great gerbils, Libyan jirds, Severtzov's jerboa (*Allactaga severtzovi*), long-eared hedgehogs (*Hemiechinus auritus*), domestic mice (*Mus musculus*) and golden hamsters (*Mesocricetus auratus*). In contrast, only the great gerbils, some Libyan jirds and golden hamsters could be infected by the LV clones or strains (Eliseev *et al*., 1980; Strelkova *et al*., 1980).

The mystery of strains with different virulence was solved only with an advent of molecular techniques in the late 1980s. The isoenzyme analysis revealed that the strains previously identified as *L. major* include 3 independent species – *L. major sensu stricto*, *L. turanica* and *L. gerbilli* (Strelkova, 1990; Strelkova *et al*., 1990*b*). These experiments also confirmed that HV and LV strains belonged to *L. major* and *L. turanica*/*L. gerbilli*, respectively. Notably, all strains isolated from humans were *L. major*, implying that *L. turanica* and *L. gerbilli* are restricted to gerbils. To sum up, all 3 abovementioned species can infect great gerbils, golden hamsters and Libyan jirds, but only *L. major* (neither *L. turanica* nor *L. gerbilli*) can infect Severtzov's jerboa, long-eared hedgehogs and domestic outbred mice.

## Communal epidemiology and ecology of *L. gerbilli*, *L. major* and *L. turanica*

### Central Asia

#### Ecology

**Fig. 1.**
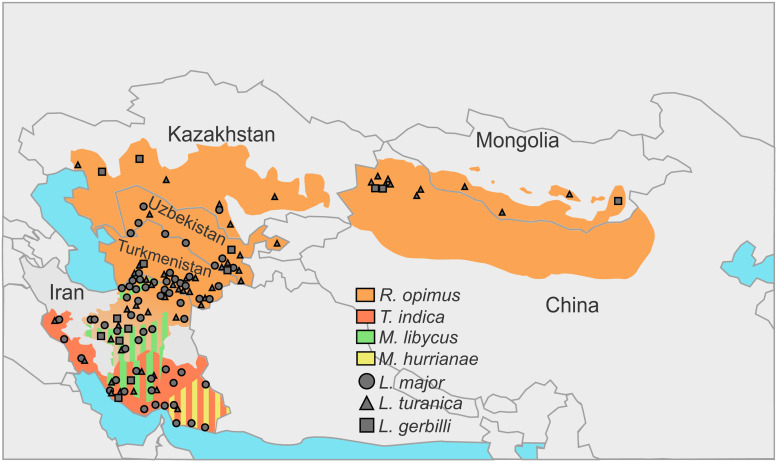
Incidences of *Leishmania* spp. in the central Asia and Middle East over the distribution areas of their predominant animal reservoirs. Stripes indicate the presence of 2 species serving as animal reservoirs in the same territory.

In the natural foci of the ZCL on the territory of Turkmenistan, Uzbekistan, Kazakhstan, Kyrgyzstan and Mongolia great gerbils are the main natural hosts of *Leishmania* spp. discussed above (Eliseev and Kellina, 1963; Dubrovsky, 1978; Eliseev and Neronov, 1997; Bruschi and Gradoni, 2018) (Fig. 1, Table 1). Parasites’ life cycle not involving *R. opimus* was shown in some rare cases (e.g. in the lower reaches of the Surkhandarya river it involves *M. libycus*) (Strelkova *et al*., 1980). Great gerbils form topical and, as a result, trophic relationships with sand flies (Abai *et al*., 2010; Akhavan *et al*., 2010*c*). They dig large and complex burrows (colonies), ideal for hatching and feeding of sand flies. Other animals [such as *M. libycus*, midday jird *Meriones meridianus*, *H. auritus* or *M. musculus* (Dubrovsky, 1978)] may cohabitate with the greater gerbils for some time usually occupying the outer parts of the colony. This significantly reduces the likelihood and intensity of sand flies’ feeding on them.

**Table 1.** ZCL in central Asia, Iran and China (data summarized for field studies)

	Reservoir host	Main vectors	*Leishmania* sp.	Mixed infections
Uzbekistan	*Rhombomys opimus*	*Phlebotomus papatasi* *Phlebotomus caucasicus* *Phlebotomus andrejevi* *Phlebotomus alexandri* *Phlebotomus mongolensis*	*Leishmania major* *Leishmania turanica* *Leishmania gerbilli*	*L. major* *+* *L. turanica* rarely *L. major* *+* *L. turanica* *+* *L. gerbilli*
Turkmenistan	*R. opimus*	*P. papatasi* *P. caucasicus* *P. andrejevi*	*L. major* *L. turanica* *L. gerbilli*	*L. major* *+* *L. turanica*
Kazakhstan South of 43°N	*R. opimus*	*P. papatasi* *P. caucasicus*	*L. major* *L. turanica*	*L. major* *+* *L. turanica*
Kazakhstan North of 43°N	*R. opimus*	*P. mongolensis*	*L. turanica* *L. gerbilli*	Not detected
Mongolia	*R. opimus*	*P. andrejevi* *P. caucasicus*	*L. turanica* *L. gerbilli*	Not detected
Iran	*R. opimus* *Meriones libycus* *Tatera indica* *Meriones hurrianae*	*P. papatasi* *Phlebotomus salehi* *P. caucasicus*/ *mongolensis* *Phlebotomus ansari*^a^	*L. major* *L. turanica* *L. gerbilli*	*L. major* *+* *L. turanica* *L. major* *+* *L. gerbilli* *L. turanica* *+* *L. gerbilli* *L. major* *+* *L. turanica* *+* *L. gerbilli*
China	*R. opimus*	*P. mongolensis* *P. andrejevi*	*L. turanica* *L. gerbilli*	Not detected

a Suspected vector.

*Note*: *Leishmania infantum* may also manifest skin symptoms, main reservoir hosts are dogs, vectors are *Phlebotomus wui*, *Phlebotomus longiductus*, *Phlebotomus alexandri*, *Phlebotomus chinensis*, *Phlebotomus sichuanensis* and *Phlebotomus smirnovi* in China; *P. alexandri*, *Phlebotomus transcaucasicus*, *Phlebotomus kandelakii*, *P. chinensis* and *P. major* s. l. in Iran; *P. longiductus* and *P. smirnovi* in Kazakhstan and Uzbekistan; *Phlebotomus turanicus* in Turkmenistan.

#### Hosts and vectors

In central Asian countries, the spectra of documented hosts and vectors vary for different *Leishmania* spp.: *L. major* has been isolated from humans, great gerbils, Libyan jirds, *Phlebotomus papatasi* and *Phlebotomus andrejevi*, while *L. turanica* and *L. gerbilli* have been found in great gerbils, *P. papatasi*, *P. andrejevi*, *Phlebotomus caucasicus*, *Phlebotomus mongolensis*, *Phlebotomus alexandri*, *Sergentomyia clydei*, and great gerbils and *P. mongolensis*, respectively (Strelkova, 1996). Out of all the identified sand fly species, only *P. papatasi* and *P. mongolensis* are anthropophilic (Killick-Kendrick, 1990; Guan *et al*., 1995). Notably, experimental coinfections of *L. major* and *L. turanica* in *P. papatasi* revealed that 2 parasite species do not outcompete each other and develop in parallel (Chajbullinova *et al*., 2012). In addition to (albeit, loose) vector specificity and host specificity, different *Leishmania* spp. inhabit somewhat different geographic areas. While *L. turanica* was found infecting *R. opimus* throughout its distribution areas (see Fig. 1 in Strelkova, 1996), *L. major* parasitizes great gerbils and Libyan jirds in more southern parts of their distribution areas predominantly in river valleys, oases, areas adjacent to the oases and foothill plains (Neronov *et al*., 1987; Strelkova *et al*., 1990*a*). Conversely, in the southern and south-western areas of Iran not populated by great gerbils, the circulation of CL is unstable confirming the role of these animals as the main reservoirs (Neronov and Farang-Azad, 1973) (Table 1, see below). *Leishmania gerbilli* is not as widespread as the other 2 species discussed above and epizootics caused by this parasite are limited to the local populations of *R. opimus* in several regions of China, Iran, Kazakhstan, Turkmenistan and Uzbekistan (Strelkova, 1996; Strelkova *et al*., 2003).

#### Infection prevalence and seasonal dynamics

The prevalence of *L. turanica* infection in *R. opimus* is high (over 50%, sometimes achieving 100% in certain populations) and fairly stable over the years. The transmission season lasts from late April to mid-October and from late May to early September for southern and northern parts of the area, respectively; it usually peaks around late June–early July and remains stable until the end of transmission period. The prevalence of *L. major* in *R. opimus* is lower than that of *L. turanica*; it peaks in July–August achieving 10–50% in different years and places. The prevalence of this parasite in *M. libycus* (usually, within the *R. opimus* distribution range) does not exceed 3–4%. The prevalence of *L. gerbilli* infection in *R. opimus* is even lower than that of *L. major* (Strelkova, 1996).

#### Coinfections, sympatric and allopatric populations

A remarkably high proportion of great gerbils in central Asia is infected by more than 1 *Leishmania* spp.; occasionally, all 3 species have been documented in the same animal (Strelkova, 1990; Strelkova *et al*., 1990*a*) (Fig. 1, Table 1). Populations of *L. gerbilli*, *L. major* and *L. turanica* are sympatric in some areas of *R. opimus* distribution (Strelkova *et al*., 2003). The human ZCL caused by *L. major* in these areas always occurs on the background of *L. turanica* or, more rarely, *L. gerbilli* infection. It has been demonstrated that coinfection with *L. turanica* facilitates *L. major* perseverance in rodents during the 6–10 months break in the sand flies-mediated transmission of these parasites in central Asia (Strelkova *et al*., 2001). In contrast, the populations of *L. turanica* in northern Kazakhstan and Mongolia are allopatric, as the Iran–Turanian part of the ZCL range is geographically isolated from the central Asian one (Shurkhal *et al*., 1985; Neronov *et al*., 1987).

The dynamics of parasite presence in sympatric populations was investigated in the cases of predominant *L. turanica* and subsidiary *L. major* in great gerbils in central Asia (Eliseev *et al*., 1991; Strelkova *et al*., 1993, 2003). Three main types of natural foci are described in *R. opimus* (Lysenko and Beljaev, 1987). The hypoendemic foci are characterized by the very low-density circulation of *L. major*; here *L. turanica* predominates throughout the year, often for several years in a row. As a consequence, human ZCL in these foci is rare. In the hyperendemic foci, the proportion of *L. major* goes up during the transmission period (May–September), in some cases achieving 50% by the end of the season. This results in a substantially higher incidence of human ZCL. *Leishmania major* in the mesoendemic foci may accumulate up to 50% prevalence in *R. opimus* for several years, while being virtually absent in certain years in-between (Eliseev *et al*., 1991; Strelkova, 1996).

Coinfections with *L. turanica* and *L. major* in great gerbils appear to be evolutionarily beneficial over the single-species infections. Indeed, while individual experimental infections with *L. major*, *L. turanica* and *L. gerbilli* lasted for 7, 15 and 18 months, respectively, the coinfection of *L. major* and *L. turanica* lasted for over 25 months (Strelkova, 1991), indirectly confirming an early observation that duration of *Leishmania* spp. infection may be comparable with the life time of a host, *R. opimus* (Shishliaeva-Matova *et al*., 1966). While infections with *L. major* alone were mostly self-healing, those with *L. gerbilli*, *L. turanica*, or coinfections with *L. major* and *L. turanica* led to chronic diseases in vast majority of cases (Strelkova, 1991).

The situation in neighbouring countries (particularly, Iran and China) is somewhat different and deserves special discussion.

### Iran

#### Hosts and vectors

The animal hosts of *L. major* are *R. opimus* in the north-eastern and central Iran, *M. libycus* in the south-western and central regions, *T. indica* in the south-west, west and south of the country and *M. hurrianae* in the south-east (Mohebali *et al*., 2004; Akhoundi *et al*., 2013). The presence of *L. turanica* was documented in great gerbils from the central and north-eastern Iran (Mohebali *et al*., 2004; Mirzaei *et al*., 2011), *M. libycus* from Fars and Esfahan provinces (Akhoundi *et al*., 2013; Asl *et al*., 2022), *Rattus norvegicus* and *T. indica* in Bushehr province (Yaghoobi-Ershadi *et al*., 2013), and short-tailed bandicoot rats (*Nesokia indica*) in the western province of Kermanshah (Hajjaran *et al*., 2009) (Fig. 1, Table 1).

#### Infection prevalence, seasonal dynamics and coinfections

The reported prevalence of single infections and coinfections with *L. major* and *L. turanica* in great gerbils varied greatly between different studies (Akhavan *et al*., 2010*b*, 2010*c*; Akhoundi *et al*., 2013; Hajjaran *et al*., 2013; Mirzaei *et al*., 2014; Asl *et al*., 2022), indicating that more studies are needed to address this question. *Leishmania gerbilli* was previously detected only in the Esfahan and Bushehr provinces in single infections and coinfections with *L. major*, *L. turanica* or both, but usually in a small fraction of animals (Mirzaei *et al*., 2011; Yaghoobi-Ershadi *et al*., 2013). A recent study reported a higher frequency of *L. gerbilli* in the central Iran: out of 162 *R. opimus* and *M. libycus* analysed, 28 and 43 were infected by *L. gerbilli* alone and *L. gerbilli* with *L. turanica*, respectively (Asl *et al*., 2022). The seasonal dynamics of *R. opimus* infection in Iran also differs from that described for the former USSR: while single *L. major* infections were observed in autumn and winter, and coinfections with *L. major* and *L. turanica* in all seasons except for summer, the single *L. turanica* infections were present throughout the year (Akhavan *et al*., 2010*a*, 2010*c*). The proven vectors of *L. major* in this country include *P. papatasi*, *Phlebotomus salehi*, *P. caucasicus* and (suspected) *Phlebotomus ansari* (Azizi *et al*., 2012; Yaghoobi-Ershadi, 2012; Maroli *et al*., 2013; Rafizadeh *et al*., 2016). The presence of *L. major*, *L. turanica*, and *L. gerbilli* in Iranian sand flies (*P. papatasi*, *P. caucasicus*, *P. mongolensis*, *P. salehi*, *Phlebotomus sergenti*, *P. ansari*, *P. alexandri* and *Sergentomyia sintoni*) was reported mostly from north-eastern and central regions (Parvizi and Ready, 2008; Bakhshi *et al*., 2013; Bordbar and Parvizi, 2014*a*; Sharbatkhori *et al*., 2014; Rafizadeh *et al*., 2016). Coinfections with *L. major* and *L. turanica* were rare and documented only in *P. papatasi* (Parvizi and Ready, 2008; Darvishi *et al*., 2015; Rafizadeh *et al*., 2016). Coinfections with *L. turanica* and *L. gerbilli* were seen in *P. papatasi* and *P. caucasicus* (Bakhshi *et al*., 2013; Darvishi *et al*., 2015). Overall, *P. papatasi* is the species, in which *L. turanica* has been found most frequently in Iran, both in single and coinfections with *L. major* or *L. gerbilli*. As its competence to support the development of *L. turanica* has also been demonstrated in the laboratory (Chajbullinova *et al*., 2012), *P. papatasi* is probably the main vector of *L. turanica* in Iran. Its vector competence for *L. gerbilli* has yet to be established.

### China

In China, both VL and CL leishmaniasis are endemic in the western and north-western areas, with a predominance of VL and rather rare cases of CL (Wang *et al*., 2010). *Leishmania major* in these regions has not been documented in rodents or humans, and human CL is caused by *L. infantum* transmitted by *Phlebotomus major wui* (Guan *et al*., 2013). *Leishmania gerbilli* has been described from desert areas inhabited by *R. opimus* since the 1960s (Wang *et al*., 1964), and *L. turanica* was first identified in *R. opimus* in mid-1990s in Xinjiang (Guan *et al*., 1995). *Phlebotomus mongolensis* and *P. andrejevi* are considered the main vectors; both are ecologically connected to *R. opimus* (Table 1). While *P. andrejevi* is scarcely captured in residential quarters, *P. mongolensis* is anthropophilic and dominates in human baits. In theory, reptilian reservoirs could also be involved in the circulation of *L. turanica* in the arid desert areas of north-western China, as this parasite has been found in 4 and 2 species of the genera *Phrynocephalus* and *Eremias*, respectively (Zhang *et al*., 2019).

In summary, the division of previously recognized as a single-species *L. major* into 3 *Leishmania* spp., of which only 1 is pathogenic for humans, facilitated revision of the earlier concepts about the epidemiology and endemicity of *L. major* in the territory that was considered well-studied.

## Pathogenicity of *L. turanica* infections for humans

In Iran, asymptomatic *Leishmania* infections prevail in the natural rodent hosts: approximately 90% of the infected *R. opimus* showed no cutaneous lesions on their earlobes (Akhavan *et al*., 2010*b*, 2010*c*; Akhoundi *et al*., 2013). Notably, among symptomatic *R. opimus*, both *L. major* and *L. turanica* were detected but not scrutinized further (Hajjaran *et al*., 2013). In line with these, single infections with *L. turanica* can cause lesions in humans too, as was demonstrated in a single study in northern Iran (Bordbar and Parvizi, 2014*b*). Subcutaneous inoculation of human volunteers with 2 strains of *L. turanica* (1 from a Mongolian great gerbil and 1 from a sand fly from Uzbekistan) resulted in mild cutaneous manifestations. The Mongolian isolate infection manifested in a small nodule, which persisted for 2 weeks and resolved with no ulceration. Infection with a strain originated from a sand fly led to a prolonged incubation period and ulceration. The lesions persisted for about 2.5 months and healed spontaneously leaving small scars (Strelkova *et al*., 1990*b*). In China, 2 healthy volunteers injected with *L. turanica* also had mild cutaneous manifestations (Guan *et al*., 1995). Collectively, *L. turanica*, a species of *Leishmania* might be categorized better as being low in pathogenicity, rather than being non-pathogenic in humans. Further studies are warranted to clarify this issue, as the number of volunteers involved in experimental infections was too small to generalize.

## Conclusions and perspectives

### Genomics

Genomes of all *Leishmania* spp. discussed above have been sequenced. The iconic one, *L. major* MHOM/IL/81/Friedlin had been sequenced earlier in 2005 (Ivens *et al*., 2005) and since then was used as a reference in numerous comparative studies (El-Sayed *et al*., 2005; Peacock *et al*., 2007; Lukeš *et al*., 2018; Zakharova *et al*., 2022). The genomes of *L. turanica* MRHO/SU/65/VL (LEM423) and *L. gerbilli* MRHO/CN/60/GERBILLI (LEM452) are also available, but they have not been scrupulously analysed yet (Warren *et al*., 2021). It goes without saying that more strains of these *Leishmania* spp. need to be sequenced and analysed in order to shed light on molecular mechanisms driving speciation in allopatric and sympatric populations of *Leishmania* spp., as well as the details defining hypoendemic, mesoendemic and hyperendemic foci of this parasite in central Asia.

### Presence of leishmaniaviruses

Another interesting and very important topic of investigation is interaction between *Leishmania* spp. and their endosymbiotic viruses. The fact that viruses can infect *Leishmania* was established half-a-century ago (Molyneux, 1974), but the real breakthrough came only after about 40 years of active investigation (Tarr *et al*., 1988; Stuart *et al*., 1992; Widmer and Dooley, 1995; Grybchuk *et al*., 2018*a*), when it was demonstrated that the presence of *Leishmania RNA virus 1* (*Leishmaniavirus 1*, LRV-1) in *Leishmania guyanensis* controls the severity of mucocutaneous leishmaniasis (Ives *et al*., 2011). The LRV-1 and LRV-2 are restricted to the New and Old World *Leishmania* spp., respectively and generally co-evolve with their hosts (Scheffter *et al*., 1995; Widmer and Dooley, 1995; Cantanhêde *et al*., 2021; Kostygov *et al*., 2021*a*). Interestingly, their elimination from *L. major* and *L. guyanensis in vitro* prompts different cellular responses (Saura *et al*., 2022). Our previous analyses of the available central Asian strains of *L. gerbilli* (*n* = 2), *L. major* (*n* = 14) and *L. turanica* (*n* = 12) revealed the presence of LRV-2 only in *L. major* with the prevalence of about 65% (Kleschenko *et al*., 2019; Kostygov *et al*., 2021*a*) corroborating findings of LRV-2 in the same parasite species in Iran and Turkey (Hajjaran *et al*., 2016; Kurt *et al*., 2019; Nalçacı *et al*., 2019; Saberi *et al*., 2020, 2022; Farrokhi-Karibozorg *et al*., 2022; Moin-Vaziri *et al*., 2022). The prevalence of *L. major* infection by LRV-2 in these studies varied between 2 and 68% and the presence of LRV-2 was confirmed in 87 out of 221 isolates of *L. major* analysed (only 1 isolate of *L. turanica* was included and reported as negative and no isolates of *L. gerbilli* were analysed).

These findings also pose an intriguing question – why *L. turanica* and *L. gerbilli* (note that conclusion about *L. gerbilli* is based on analysis of a very few isolates) are not infected with LRV-2? This is unlikely to be a virus-related feature, as LRVs were shown to be able to cross species barriers and even infect trypanosomatids of other genera (Grybchuk *et al*., 2018*b*). This is also unlikely to be determined by the vector, because all 3 *Leishmania* spp. are transmitted by the same or closely related species of sand flies (Strelkova, 1996). The most parsimonious explanation is that *L. gerbilli* and *L. turanica* are naturally resistant against LRV-2. If this is really the case, analysis of additional strains of *L. gerbilli* and *L. turanica* will help us to discover genetic elements making them resilient to viral infections possibly providing new tools for treatment of leishmaniases. It is also important to consider how LRVs are transmitted. While to the best of our knowledge no experimental studies were performed on LRV-2s, in the cases of LRV-1 and New World *Leishmania* spp. it has been shown that viruses are transmitted horizontally *via* extracellular vesicles (Atayde *et al*., 2019; Olivier and Zamboni, 2020).

In conclusion, we are now on the verge of new and exciting findings fuelled by recent development in sequencing technologies that may dramatically expand our knowledge about parasites causing leishmaniasis and change the way this disease is diagnosed and treated.

## Author's contributions

Writing and revision – all authors.

## References

[ref1] Abai MR, Oshaghi MA, Tajedin L, Rassi Y and Akhavan AA (2010) Geographical distribution and ecological features of the great gerbil subspecies in the main zoonotic cutaneous leishmaniasis foci in Iran. Asian Pacific Journal of Tropical Medicine 3, 800–803.

[ref2] Akhavan A, Yaghoobi-Ershadi M, Mirhendi H, Alimohammadian M, Rassi Y, Shareghi N, Jafari R, Arandian M, Abdoli H, Ghanei M, Jalali-Zand N and Khamesipour A (2010 *a*) Molecular epizootiology of rodent leishmaniasis in a hyperendemic area of Iran. Iranian Journal of Public Health 39, 1–7. PMC3468974 23112983

[ref3] Akhavan AA, Mirhendi H, Khamesipour A, Alimohammadian MH, Rassi Y, Bates P, Kamhawi S, Valenzuela JG, Arandian MH, Abdoli H, Jalali-zand N, Jafari R, Shareghi N, Ghanei M and Yaghoobi-Ershadi MR (2010 *b*) *Leishmania* species: detection and identification by nested PCR assay from skin samples of rodent reservoirs. Experimental Parasitology 126, 552–556. 20566364 10.1016/j.exppara.2010.06.003 PMC2939322

[ref4] Akhavan AA, Yaghoobi-Ershadi MR, Khamesipour A, Mirhendi H, Alimohammadian MH, Rassi Y, Arandian MH, Jafari R, Abdoli H, Shareghi N, Ghanei M and Jalali-zand N (2010 *c*) Dynamics of *Leishmania* infection rates in *Rhombomys opimus* (Rodentia: Gerbillinae) population of an endemic focus of zoonotic cutaneous leishmaniasis in Iran. Bulletin de la Societe de Pathologie Exotique 103, 84–89. 20390397 10.1007/s13149-010-0044-1

[ref5] Akhoundi M, Mohebali M, Asadi M, Mahmodi MR, Amraei K and Mirzaei A (2013) Molecular characterization of *Leishmania* spp. in reservoir hosts in endemic foci of zoonotic cutaneous leishmaniasis in Iran. Folia Parasitologica 60, 218–224. 23951928 10.14411/fp.2013.024

[ref6] Akilov OE, Khachemoune A and Hasan T (2007) Clinical manifestations and classification of Old World cutaneous leishmaniasis. International Journal of Dermatology 46, 132–142. 17269962 10.1111/j.1365-4632.2007.03154.x

[ref7] Alborzi A, Pouladfar GR, Fakhar M, Motazedian MH, Hatam GR and Kadivar MR (2008) Isolation of *Leishmania tropica* from a patient with visceral leishmaniasis and disseminated cutaneous leishmaniasis, southern Iran. American Journal of Tropical Medicine and Hygiene 79, 435–437. 18784238

[ref8] Alvar J, Velez ID, Bern C, Herrero M, Desjeux P, Cano J, Jannin J and den Boer M (2012) Leishmaniasis worldwide and global estimates of its incidence. PLoS ONE 7, e35671. 22693548 10.1371/journal.pone.0035671 PMC3365071

[ref9] Asl FG, Mohebali M, Jafari R, Akhavan AA, Shirzadi MR, Zarei Z, Fadaei R, Ramezanpour J, Hassanpour G, Izadi S, Hajjaran H and Elikaee S (2022) *Leishmania* spp. infection in *Rhombomys opimus* and *Meriones libycus* as main reservoirs of zoonotic cutaneous leishmaniasis in central parts of Iran: progress and implications in health policy. Acta Tropica 226, 106267. 34890542 10.1016/j.actatropica.2021.106267

[ref10] Atayde VD, da Silva Lira Filho A, Chaparro V, Zimmermann A, Martel C, Jaramillo M and Olivier M (2019) Exploitation of the *Leishmania* exosomal pathway by *Leishmania RNA virus* 1. Nature Microbiology 4, 714–723. 10.1038/s41564-018-0352-y 30692670

[ref11] Azizi K, Fakoorziba MR, Jalali M and Moemenbellah-Fard MD (2012) First molecular detection of *Leishmania major* within naturally infected *Phlebotomus salehi* from a zoonotic cutaneous leishmaniasis focus in southern Iran. Tropical Biomedicine 29, 1–8. 22543597

[ref12] Bakhshi H, Oshaghi MA, Abai MR, Rassi Y, Akhavan AA, Sheikh Z, Mohtarami F, Saidi Z, Mirzajani H and Anjomruz M (2013) Molecular detection of *Leishmania* infection in sand flies in border line of Iran–Turkmenistan: restricted and permissive vectors. Experimental Parasitology 135, 382–387. 23933280 10.1016/j.exppara.2013.07.020

[ref13] Ben-Shimol S, Sagi O, Horev A, Avni YS, Ziv M and Riesenberg K (2016) Cutaneous leishmaniasis caused by *Leishmania infantum* in southern Israel. Acta Parasitologica 61, 855–858. 27787222 10.1515/ap-2016-0118

[ref14] Bordbar A and Parvizi P (2014 *a*) High density of *Leishmania major* and rarity of other mammals' *Leishmania* in zoonotic cutaneous leishmaniasis foci, Iran. Tropical Medicine & International Health 19, 355–363. 24382378 10.1111/tmi.12258

[ref15] Bordbar A and Parvizi P (2014 *b*) High infection frequency, low diversity of *Leishmania major* and first detection of *Leishmania turanica* in human in northern Iran. Acta Tropica 133, 69–72. 24530890 10.1016/j.actatropica.2014.01.016

[ref16] Borovsky PF (1898) On sart sore. Russian Military Medical Journal, CXCV, CXCV(11), 925–941 (in Russian).

[ref17] Bray RS, Ashford RW and Bray MA (1973) The parasite causing cutaneous leishmaniasis in Ethiopia. Transactions of the Royal Society of Tropical Medicine and Hygiene 67, 345–348. 4778189 10.1016/0035-9203(73)90111-9

[ref18] Bruschi F and Gradoni L (2018) The Leishmaniases: Old Neglected Tropical Diseases. Cham, Switzerland: Springer.

[ref19] Cantanhêde LM, Mata-Somarribas C, Chourabi K, Pereira da Silva G, Dias das Chagas B, de Oliveira RPL, Cortes Boite M and Cupolillo E (2021) The maze pathway of coevolution: a critical review over the *Leishmania* and its endosymbiotic history. Genes 12, 657. 33925663 10.3390/genes12050657 PMC8146029

[ref20] Chajbullinova A, Votýpka J, Sádlová J, Kvapilová K, Seblová V, Kreisinger J, Jirků M, Sanjoba C, Gantuya S, Matsumoto Y and Volf P (2012) The development of *Leishmania turanica* in sand flies and competition with *L. major*. Parasites & Vectors 5, 219. 23031344 10.1186/1756-3305-5-219 PMC3484061

[ref21] Charyyeva A, Çetinkaya U, Özkan B, Şahin S, Yaprak N, Şahin I, Yurchenko V and Kostygov AY (2021) Genetic diversity of *Leishmania tropica*: unexpectedly complex distribution pattern. Acta Tropica 218, 105888. 33713626 10.1016/j.actatropica.2021.105888

[ref22] Cunningham DD (1885) On the presence of peculiar parasitic organisms in the tissue of a specimen of Delhi boil. Scientific Memoirs by Medical Officers of the Army of India 1, 21.

[ref23] Darvishi M, Yaghoobi-Ershadi MR, Shahbazi F, Akhavan AA, Jafari R, Soleimani H, Yaghoobi-Ershadi N, Khajeian M, Darabi H and Arandian MH (2015) Epidemiological study on sand flies in an endemic focus of cutaneous leishmaniasis, Bushehr city, southwestern Iran. Frontiers in Public Health 3, 14. 25699245 10.3389/fpubh.2015.00014 PMC4313593

[ref24] Donovan C (1903) A possible cause of kala-azar. Indian Medical Gazette 38, 478. PMC5150752 29002982

[ref25] Dubrovsky YA (1978) Gerbils and Natural Foci of Cutaneous Leishmaniasis. Moscow: Nauka (in Russian).

[ref27] Elfari M, Schnur LF, Strelkova MV, Eisenberger CL, Jacobson RL, Greenblatt CL, Presber W and Schönian G (2005) Genetic and biological diversity among populations of *Leishmania major* from central Asia, the Middle East and Africa. Microbes and Infection 7, 93–103. 15716069 10.1016/j.micinf.2004.09.010

[ref28] Eliseev LN and Kellina OI (1963) Cutaneous leishmaniasis in Afghanistan. Meditsinskaia Parazitologiia 32, 728–735 (in Russian). 14159831

[ref29] Eliseev LN and Neronov VM (1997) Landscape-ecological analysis of distribution of *Rhombomys opimus* in Afghanistan, Iran and Pakistan. Biology Bulletin Reviews 117, 602–623 (in Russian).

[ref30] Eliseev LN, Strelkova MV and Passova OM (1980) Interrelationship characteristics of *Leishmania major* with various mammalian species. Meditsinskaia Parazitologiia 49, 42–49 (in Russian). 7207384

[ref31] Eliseev LN, Strelkova MV and Zherikhina II (1991) The characteristics of the epidemic activation of a natural focus of zoonotic cutaneous leishmaniasis in places with a sympatric dissemination of *Leishmania major*, *L. turanica* and *L. gerbilli*. Meditsinskaia Parazitologiia, 24–29 (in Russian). 1837582

[ref26] El-Sayed NM, Myler PJ, Blandin G, Berriman M, Crabtree J, Aggarwal G, Caler E, Renauld H, Worthey EA, Hertz-Fowler C, Ghedin E, Peacock C, Bartholomeu DC, Haas BJ, Tran AN, Wortman JR, Alsmark UC, Angiuoli S, Anupama A, Badger J, Bringaud F, Cadag E, Carlton JM, Cerqueira GC, Creasy T, Delcher AL, Djikeng A, Embley TM, Hauser C, Ivens AC, Kummerfeld SK, Pereira-Leal JB, Nilsson D, Peterson J, Salzberg SL, Shallom J, Silva JC, Sundaram J, Westenberger S, White O, Melville SE, Donelson JE, Andersson B, Stuart KD and Hall N (2005) Comparative genomics of trypanosomatid parasitic protozoa. Science 309, 404–409. 16020724 10.1126/science.1112181

[ref32] Farrokhi-Karibozorg M, Ghayour-Najafabadi Z, Hejazi SH, Ataei-Pirkooh A, Mohebali M, Teimouri P and Hajjaran H (2022) Molecular identification of *Leishmania RNA virus* in cutaneous leishmaniasis patients and rodent reservoirs in Isfahan province, Iran. Infection Genetics and Evolution 98, 105222. 10.1016/j.meegid.2022.105222 35066166

[ref33] Ghatee MA, Taylor WR and Karamian M (2020) The geographical distribution of cutaneous leishmaniasis causative agents in Iran and its neighboring countries, a review. Frontiers in Public Health 8, 11. 32133334 10.3389/fpubh.2020.00011 PMC7039857

[ref34] Ghawar W, Toumi A, Snoussi MA, Chlif S, Zaatour A, Boukthir A, Hamida NB, Chemkhi J, Diouani MF and Ben-Salah A (2011) *Leishmania major* infection among *Psammomys obesus* and *Meriones shawi*: reservoirs of zoonotic cutaneous leishmaniasis in Sidi Bouzid (central Tunisia). Vector Borne and Zoonotic Diseases 11, 1561–1568. 21919726 10.1089/vbz.2011.0712 PMC3263488

[ref35] Grybchuk D, Kostygov AY, Macedo DH, d'Avila-Levy CM and Yurchenko V (2018 *a*) RNA viruses in trypanosomatid parasites: a historical overview. Memorias do Instituto Oswaldo Cruz 113, e170487. 29513877 10.1590/0074-02760170487 PMC5851034

[ref36] Grybchuk D, Kostygov AY, Macedo DH, Votýpka J, Lukeš J and Yurchenko V (2018 *b*) RNA viruses in *Blechomonas* (Trypanosomatidae) and evolution of *Leishmaniavirus*. mBio 9, e01932–e01918. 10.1128/mBio.01932-18 PMC6191543 30327446

[ref37] Guan LR, Yang YQ, Qu JQ and Shen WX (1995) Discovery and study of *Leishmania turanica* for the first time in China. Bulletin of the World Health Organization 73, 667–672. 8846493 PMC2486811

[ref38] Guan LR, Yang YQ, Qu JQ, Ren HY and Chai JJ (2013) Discovery and study of cutaneous leishmaniasis in Karamay of Xinjiang, West China. Infectious Diseases of Poverty 2, 20. 24010525 10.1186/2049-9957-2-20 PMC3856449

[ref39] Hajjaran H, Mohebali M, Alimoradi S, Abaei MR and Edrissian GH (2009). Isolation and characterization of pathogenic *Leishmania turanica* from *Nesokia indica* (Rodentia, Muridae) by PCR-RFLP and ITS1 sequencing in Iran. Transactions of the Royal Society of Tropical Medicine and Hygiene 103, 1177–1179. 18829057 10.1016/j.trstmh.2008.08.016

[ref40] Hajjaran H, Mohebali M, Abaei MR, Oshaghi MA, Zarei Z, Charehdar S, Mirjalali H, Sharifdini M and Teimouri A (2013) Natural infection and phylogenetic classification of *Leishmania* spp. infecting *Rhombomys opimus*, a primary reservoir host of zoonotic cutaneous leishmaniasis in northeast Iran. Transactions of the Royal Society of Tropical Medicine and Hygiene 107, 550–557. 23868742 10.1093/trstmh/trt060

[ref41] Hajjaran H, Mahdi M, Mohebali M, Samimi-Rad K, Ataei-Pirkooh A, Kazemi-Rad E, Naddaf SR and Raoofian R (2016) Detection and molecular identification of *Leishmania RNA virus* (LRV) in Iranian *Leishmania* species. Archives of Virology 161, 3385–3390. 27604119 10.1007/s00705-016-3044-z

[ref42] Hoare CA (1938) Early discoveries regarding the parasite of oriental sore (with an English translation of the memoir by P. F. Borovsky: ‘On Sart sore’, 1898). Transactions of the Royal Society of Tropical Medicine and Hygiene 32, 67–92.

[ref43] Ivens AC, Peacock CS, Worthey EA, Murphy L, Aggarwal G, Berriman M, Sisk E, Rajandream MA, Adlem E, Aert R, Anupama A, Apostolou Z, Attipoe P, Bason N, Bauser C, Beck A, Beverley SM, Bianchettin G, Borzym K, Bothe G, Bruschi CV, Collins M, Cadag E, Ciarloni L, Clayton C, Coulson RM, Cronin A, Cruz AK, Davies RM, De Gaudenzi J, Dobson DE, Duesterhoeft A, Fazelina G, Fosker N, Frasch AC, Fraser A, Fuchs M, Gabel C, Goble A, Goffeau A, Harris D, Hertz-Fowler C, Hilbert H, Horn D, Huang Y, Klages S, Knights A, Kube M, Larke N, Litvin L, Lord A, Louie T, Marra M, Masuy D, Matthews K, Michaeli S, Mottram JC, Muller-Auer S, Munden H, Nelson S, Norbertczak H, Oliver K, O'Neil S, Pentony M, Pohl TM, Price C, Purnelle B, Quail MA, Rabbinowitsch E, Reinhardt R, Rieger M, Rinta J, Robben J, Robertson L, Ruiz JC, Rutter S, Saunders D, Schafer M, Schein J, Schwartz DC, Seeger K, Seyler A, Sharp S, Shin H, Sivam D, Squares R, Squares S, Tosato V, Vogt C, Volckaert G, Wambutt R, Warren T, Wedler H, Woodward J, Zhou S, Zimmermann W, Smith DF, Blackwell JM, Stuart KD, Barrell B and Myler PJ (2005) The genome of the kinetoplastid parasite, *Leishmania major*. Science (New York, N.Y.) 309, 436–442. 16020728 10.1126/science.1112680 PMC1470643

[ref44] Ives A, Ronet C, Prevel F, Ruzzante G, Fuertes-Marraco S, Schutz F, Zangger H, Revaz-Breton M, Lye LF, Hickerson SM, Beverley SM, Acha-Orbea H, Launois P, Fasel N and Masina S (2011) *Leishmania* RNA virus controls the severity of mucocutaneous leishmaniasis. Science (New York, N.Y.) 331, 775–778. 21311023 10.1126/science.1199326 PMC3253482

[ref45] Kellina OI (1965) A comparative study of the virulence of *Leishmania tropica major* strains. Meditsinskaia Parazitologiia 34, 309–317 (in Russian). 5869240

[ref46] Kellina OI, Passova OM and Alekseev AN (1981) Experimental proof of the heterogeneous composition of natural *Leishmania major* populations for the virulence trait. Meditsinskaia Parazitologiia 50, 4–11 (in Russian). 7322024

[ref47] Killick-Kendrick R (1990) Phlebotomine vectors of the leishmaniases: a review. Medical and Veterinary Entomology 4, 1–24. 2132963 10.1111/j.1365-2915.1990.tb00255.x

[ref48] Kleschenko Y, Grybchuk D, Matveeva NS, Macedo DH, Ponirovsky EN, Lukashev AN and Yurchenko V (2019) Molecular characterization of *Leishmania RNA virus* 2 in *Leishmania major* from Uzbekistan. Genes 10, e830. 10.3390/genes10100830 PMC6826456 31640177

[ref49] Kostygov AY, Grybchuk D, Kleschenko Y, Chistyakov DS, Lukashev AN, Gerasimov ES and Yurchenko V (2021 *a*) Analyses of *Leishmania*-LRV co-phylogenetic patterns and evolutionary variability of viral proteins. Viruses 13, 2305. 34835111 10.3390/v13112305 PMC8624691

[ref50] Kostygov AY, Karnkowska A, Votýpka J, Tashyreva D, Maciszewski K, Yurchenko V and Lukeš J (2021 *b*) Euglenozoa: taxonomy, diversity and ecology, symbioses and viruses. Open Biology 11, 200407. 33715388 10.1098/rsob.200407 PMC8061765

[ref51] Kozevnikov PV (1963) Two nosological forms of cutaneous leishmaniasis. American Journal of Tropical Medicine and Hygiene 12, 719–724. 14070762 10.4269/ajtmh.1963.12.719

[ref52] Kurt O, Mansur N, Çavuş I, Özcan O, Batir MB, Gündüz C, Sezerman OU and Özbilgın A (2019) First report and *in silico* analysis of *Leishmania virus* (LRV2) identified in an autochthonous *Leishmania major* isolate in Turkey. The New Microbiologica 42, 64–67. 30671580

[ref53] Latyshev NI and Krukova AP (1941) On the epidemiology of the cutaneous leishmaniasis. The cutaneous leishmaniasis as zoonotic disease of wild rodents in Turkmenia. In *Proceedings of the Military Medical Academy of the Red Army*. Leningrad, Vol. 25, pp. 229–242 (in Russian).

[ref54] Lavrova MI, Kellina OI, Passova OM and Shuikina EE (1973) Some mechanisms of distribution of highly virulent strains of *Leishmania tropica major* isolated from the large gerbil (*Rhombomys opimus* Licht.) in Karshinsk steppe. Meditsinskaia Parazitologiia 42, 58–61 (in Russian). 4281857

[ref55] Leishman WB (1903) On the possibility of the occurrence of trypanosomiasis in India. British Medical Journal 1, 1252–1254. 16789342

[ref56] Lühe M (1906) Die im Blute schmarotzenden Protozoen und ihre nächsten Verwandten. In Mense CA (ed). Handbuch der Tropenkrankheiten, Leipzig: J. A. Barth, Vol. 3. pp. 69–268.

[ref57] Lukeš J, Mauricio IL, Schönian G, Dujardin JC, Soteriadou K, Dedet JP, Kuhls K, Tintaya KW, Jirků M, Chocholová E, Haralambous C, Pratlong F, Oborník M, Horák A, Ayala FJ and Miles MA (2007) Evolutionary and geographical history of the *Leishmania donovani* complex with a revision of current taxonomy. Proceedings of the National Academy of Sciences of the USA 104, 9375–9380. 17517634 10.1073/pnas.0703678104 PMC1890502

[ref58] Lukeš J, Butenko A, Hashimi H, Maslov DA, Votýpka J and Yurchenko V (2018) Trypanosomatids are much more than just trypanosomes: clues from the expanded family tree. Trends in Parasitology 34, 466–480. 29605546 10.1016/j.pt.2018.03.002

[ref59] Lysenko AJ and Beljaev AE (1987) Quantitative approaches to epidemiology. In Peters W and Killick-Kendrick R (eds), The Leishmaniases in Biology and Medicine, Vol. 1. Biology and Epidemiology. London: Academic Press, pp. 263–290.

[ref60] Mann S, Frasca K, Scherrer S, Henao-Martinez AF, Newman S, Ramanan P and Suarez JA (2021) A review of leishmaniasis: current knowledge and future directions. Current Tropical Medicine Reports 8, 121–132. 33747716 10.1007/s40475-021-00232-7 PMC7966913

[ref61] Maroli M, Feliciangeli MD, Bichaud L, Charrel RN and Gradoni L (2013) Phlebotomine sandflies and the spreading of leishmaniases and other diseases of public health concern. Medical and Veterinary Entomology 27, 123–147. 22924419 10.1111/j.1365-2915.2012.01034.x

[ref62] Mirzaei A, Rouhani S, Taherkhani H, Farahmand M, Kazemi B, Hedayati M, Baghaei A, Davari B and Parvizi P (2011) Isolation and detection of *Leishmania* species among naturally infected *Rhombomis opimus*, a reservoir host of zoonotic cutaneous leishmaniasis in Turkemen Sahara, North East of Iran. Experimental Parasitology 129, 375–380. 21945269 10.1016/j.exppara.2011.08.020

[ref63] Mirzaei A, Schweynoch C, Rouhani S, Parvizi P and Schönian G (2014) Diversity of *Leishmania* species and of strains of *Leishmania major* isolated from desert rodents in different foci of cutaneous leishmaniasis in Iran. Transactions of the Royal Society of Tropical Medicine and Hygiene 108, 502–512. 24980555 10.1093/trstmh/tru085

[ref64] Mohebali M, Javadian E, Yaghoobi-Ershadi MR, Akhavan AA, Hajjaran H and Abaei MR (2004) Characterization of *Leishmania* infection in rodents from endemic areas of the Islamic Republic of Iran. Eastern Mediterranean Health Journal 10, 591–599. 16335651

[ref65] Moin-Vaziri V, Zare F, Seyyed Tabaei SJ, Saberi R and Hajjaran H (2022) Successful isolation of *Leishmania RNA Virus* (LRV) from *Leishmania major* in a cutaneous leishmaniasis focus in central Iran: an update on cases. Acta Parasitologica 67, 1290–1298. 35773567 10.1007/s11686-022-00575-9 PMC9245859

[ref66] Molyneux DH (1974) Virus-like particles in *Leishmania* parasites. Nature 249, 588–589. 4834085 10.1038/249588a0

[ref67] Nalçacı M, Karakuş M, Yilmaz B, Demir S, Özbilgin A, Özbel Y and Töz S (2019) Detection of *Leishmania RNA virus* 2 in *Leishmania* species from Turkey. Transactions of the Royal Society of Tropical Medicine and Hygiene 113, 410–417. 31034027 10.1093/trstmh/trz023

[ref68] Neronov VM and Farang-Azad A (1973) Cutaneous leishmaniasis in Iran, its reservoirs and vectors (a review of the literature). Meditsinskaia Parazitologiia 42, 666–674 (in Russian). 4593360

[ref69] Neronov VM, Strelkova MV, Shurkhal AA, Luschekina AA and Artemyev MM (1987) Natural focality of zoonotic cutaneous leishmaniasis in the Mongolian People's Republic; results and objectives of integrated research. Folia Parasitologica 34, 1–9. 3583127

[ref70] Olivier M and Zamboni DS (2020) *Leishmania* (*Viannia*) *guyanensis*, LRV1 virus and extracellular vesicles: a dangerous trio influencing the faith of immune response during muco-cutaneous leishmaniasis. Current Opinion in Immunology 66, 108–113. 32877837 10.1016/j.coi.2020.08.004

[ref71] Pace D (2014) Leishmaniasis. The Journal of Infection 69, S10–S18. 25238669 10.1016/j.jinf.2014.07.016

[ref72] Parvizi P and Ready PD (2008) Nested PCRs and sequencing of nuclear ITS-rDNA fragments detect three *Leishmania* species of gerbils in sandflies from Iranian foci of zoonotic cutaneous leishmaniasis. Tropical Medicine & International Health 13, 1159–1171. 18631311 10.1111/j.1365-3156.2008.02121.x

[ref73] Parvizi P, Moradi G, Akbari G, Farahmand M, Ready PD, Piazak N, Assmar M and Amirkhani A (2008) PCR detection and sequencing of parasite ITS-rDNA gene from reservoirs host of zoonotic cutaneous leishmaniasis in central Iran. Parasitology Research 103, 1273–1278. 18791741 10.1007/s00436-008-1124-z

[ref74] Peacock CS, Seeger K, Harris D, Murphy L, Ruiz JC, Quail MA, Peters N, Adlem E, Tivey A, Aslett M, Kerhornou A, Ivens A, Fraser A, Rajandream MA, Carver T, Norbertczak H, Chillingworth T, Hance Z, Jagels K, Moule S, Ormond D, Rutter S, Squares R, Whitehead S, Rabbinowitsch E, Arrowsmith C, White B, Thurston S, Bringaud F, Baldauf SL, Faulconbridge A, Jeffares D, Depledge DP, Oyola SO, Hilley JD, Brito LO, Tosi LR, Barrell B, Cruz AK, Mottram JC, Smith DF and Berriman M (2007) Comparative genomic analysis of three *Leishmania* species that cause diverse human disease. Nature Genetics 39, 839–847. 17572675 10.1038/ng2053 PMC2592530

[ref75] Rafizadeh S, Saraei M, Abaei MR, Oshaghi MA, Mohebali M, Peymani A, Naserpour-Farivar T, Bakhshi H and Rassi Y (2016) Molecular detection of *Leishmania major* and *L. turanica* in *Phlebotomus papatasi* and first natural infection of *P. salehi* to *L. major* in north-east of Iran. Journal of Arthropod-Borne Diseases 10, 141–147. 27308272 PMC4906753

[ref76] Rassi Y, Jalati M, Javadian E and Moatazadian MH (2001) Confirmation of *Meriones libycus* (Rodentia; Gerbillidae) as the main reservoir host of zoonotic cutaneous leishmaniasis in Arsanjan, Fars province, South of Iran (1999–2000). Iranian Journal of Public Health 30, 143–144.

[ref77] Saberi R, Fakhar M, Hajjaran H, Ataei-Pirkooh A, Mohebali M, Taghipour N, Ziaei Hezarjaribi H, Moghadam Y and Bagheri A (2020) Presence and diversity of *Leishmania RNA virus* in an old zoonotic cutaneous leishmaniasis focus, northeastern Iran: haplotype and phylogenetic based approach. International Journal of Infectious Diseases 101, 6–13. 32947050 10.1016/j.ijid.2020.08.033

[ref78] Saberi R, Fakhar M, Hajjaran H, Abbaszadeh Afshar MJ, Mohebali M, Hezarjaribi HZ, Moghadam Y and Sharbatkhori M (2022) *Leishmania RNA virus* 2 (LRV2) exacerbates dermal lesions caused by *Leishmania major* and comparatively unresponsive to meglumine antimoniate treatment. Experimental Parasitology 241, 108340. 35932908 10.1016/j.exppara.2022.108340

[ref79] Saura A, Zakharova A, Klocek D, Gerasimov ES, Butenko A, Macedo DH, Servienė E, Zagirova D, Meshcheryakova A, Rogozin IB, Serva S, Kostygov AY and Yurchenko V (2022) Elimination of LRVs elicits different responses in *Leishmania* spp. mSphere 7, e0033522. 35943162 10.1128/msphere.00335-22 PMC9429963

[ref80] Scheffter SM, Ro YT, Chung IK and Patterson JL (1995) The complete sequence of *Leishmania RNA virus* LRV2-1, a virus of an Old World parasite strain. Virology 212, 84–90. 7676652 10.1006/viro.1995.1456

[ref81] Schnur LF (1987) On the clinical manifestations and parasites of Old World leishmaniasis and *Leishmania tropica* causing visceral leishmaniasis. In Hart DT (ed.), Leishmaniasis: The Current Status and New Strategies for Control. New York: Springer Science+Business Media, Vol. 171, pp. 939–943.

[ref82] Sharbatkhori M, Spotin A, Taherkhani H, Roshanghalb M and Parvizi P (2014) Molecular variation in *Leishmania* parasites from sandflies species of a zoonotic cutaneous leishmaniasis in northeast of Iran. Journal of Vector Borne Diseases 51, 16–21. 24717197

[ref83] Shishliaeva-Matova ZS, Ni G and Zviagintseva TV (1966) Pathogenicity of leptomonad strains isolated from sandflies in natural foci of zoonotic cutaneous leishmaniasis in Uzbekistan. Meditsinskaia Parazitologiia 35, 266–270 (in Russian). 4238257

[ref84] Shurkhal AV, Strelkova MV, Passova OM, Rakitskaia TA and Podogas AV (1985) Genetic characteristics of *Leishmania* strains isolated from gerbils in the Mongolian People's Republic. Meditsinskaia Parazitologiia 38–44 (in Russian). 3864006

[ref85] Strelkova MV (1990) The isoenzyme identification and pathogenic characteristics of clones of *Leishmania major*, *L*. sp. nov. and *L. gerbilli*. Meditsinskaia Parazitologiia 9–13 (in Russian). 2290404

[ref86] Strelkova MV (1991) Susceptibility to and the characteristics of the course of experimental leishmaniasis in different species of mammals infected with *Leishmania major*, *L. turanica* and *L. gerbilli*. Meditsinskaia Parazitologiia 35–39 (in Russian). 2067472

[ref87] Strelkova MV (1996) Progress in studies on central Asian foci of zoonotic cutaneous leishmaniasis: a review. Folia Parasitologica 43, 1–6. 8682405

[ref88] Strelkova MV, Eliseev LN, Passova OM and Valevich TA (1980) Characteristics of the relationship between gerbils and *Leishmania major*. Meditsinskaia Parazitologiia 49, 35–42 (in Russian). 7393132

[ref89] Strelkova MV, Shurkhal AV, Eliseev LN, Kellina OI, Rakitskaia TA, Zviagintseva TV, Peters U and Evans D (1990 *a*) The isoenzyme identification and pathogenic characteristics of the *Leishmania* isolated in natural foci of cutaneous leishmaniasis in the USSR. Meditsinskaia Parazitologiia 43–48 (in Russian). 2266904

[ref90] Strelkova MV, Shurkhal AV, Kellina OI, Eliseev LN, Evans DA, Peters W, Chapman CJ, Le Blancq SM and van Eys GJ (1990 *b*) A new species of *Leishmania* isolated from the great gerbil *Rhombomys opimus*. Parasitology 101, 327–335. 2092289 10.1017/s0031182000060510

[ref91] Strelkova MV, Eliseev LN, Ponirovskii EN, Erokhin PI, Rakitskaia TA, Valevich TA, Sysoev VV, Allenov VA, Adamishina TA and Dergacheva TI (1993) The isoenzyme identification of *Leishmania* isolates taken from greater gerbils, sandflies and human patients in foci of zoonotic cutaneous leishmaniasis in Turkmenistan. Meditsinskaia Parazitologiia 34–37 (in Russian). 8127269

[ref92] Strelkova MV, Eliseev LN, Ponirovsky EN, Dergacheva TI, Annacharyeva DK, Erokhin PI and Evans DA (2001) Mixed leishmanial infections in *Rhombomys opimus*: a key to the persistence of *Leishmania major* from one transmission season to the next. Annals of Tropical Medicine & Parasitology 95, 811–819. 11784435 10.1080/00034980120111154

[ref93] Strelkova MV, Shendrik AG, El Fari M and Schönian G (2003) Ecology and the genetic structure of sympatric *Leishmania* species circulating in the intra-continental deserts of the south Palaearctic region. Meditsinskaia Parazitologiia 2003, 12–18 (in Russian). 14564836

[ref94] Strelkova MV, Ponirovsky EN, Morozov EN, Zhirenkina EN, Razakov SA, Kovalenko DA, Schnur LF and Schönian G (2015) A narrative review of visceral leishmaniasis in Armenia, Azerbaijan, Georgia, Kazakhstan, Kyrgyzstan, Tajikistan, Turkmenistan, Uzbekistan, the Crimean Peninsula and Southern Russia. Parasites & Vectors 8, 330. 26077778 10.1186/s13071-015-0925-z PMC4474452

[ref95] Stuart KD, Weeks R, Guilbride L and Myler PJ (1992) Molecular organization of *Leishmania RNA virus* 1. Proceedings of the National Academy of Sciences of the USA 89, 8596–8600. 1382295 10.1073/pnas.89.18.8596 PMC49967

[ref96] Tarr PI, Aline RF Jr., Smiley BL, Scholler J, Keithly J and Stuart K (1988) LR1: a candidate RNA virus of *Leishmania*. Proceedings of the National Academy of Sciences of the USA 85, 9572–9575. 3200841 10.1073/pnas.85.24.9572 PMC282800

[ref97] Torres-Guerrero E, Quintanilla-Cedillo MR, Ruiz-Esmenjaud J and Arenas R (2017) Leishmaniasis: a review. F1000Research 6, 750. 28649370 10.12688/f1000research.11120.1 PMC5464238

[ref98] Wang J, Qu J and Guan L (1964) A study of *Leishmania* parasite of big gerbil in Northwest China. Acta Parasitologica Sinica 1, 105–117.

[ref99] Wang JY, Gao CH, Yang YT, Chen HT, Zhu XH, Lv S, Chen SB, Tong SX, Steinmann P, Ziegelbauer K and Zhou XN (2010) An outbreak of the desert sub-type of zoonotic visceral leishmaniasis in Jiashi, Xinjiang Uygur Autonomous Region, People's Republic of China. Parasitology International 59, 331–337. 20434585 10.1016/j.parint.2010.04.002

[ref100] Warren WC, Akopyants NS, Dobson DE, Hertz-Fowler C, Lye LF, Myler PJ, Ramasamy G, Shanmugasundram A, Silva-Franco F, Steinbiss S, Tomlinson C, Wilson RK and Beverley SM (2021) Genome assemblies across the diverse evolutionary spectrum of *Leishmania* protozoan parasites. Microbiology Resource Announcements 10, e0054521. 34472979 10.1128/MRA.00545-21 PMC8411921

[ref101] WHO (2022) Leishmaniasis. Available at https://www.who.int/en/news-room/fact-sheets/detail/leishmaniasis (Accessed 11.11.2022).

[ref102] Widmer G and Dooley S (1995) Phylogenetic analysis of *Leishmania RNA virus* and *Leishmania* suggests ancient virus-parasite association. Nucleic Acids Research 23, 2300–2304. 7610059 10.1093/nar/23.12.2300 PMC307021

[ref103] Wright JH (1903) Protozoa in a case of tropical ulcer (‘Delhi sore’). The Journal of Medical Research 10, 472–482. 19971589 PMC2105980

[ref104] Yaghoobi-Ershadi M (2012) Phlebotomine sand flies (Diptera: Psychodidae) in Iran and their role on *Leishmania* transmission. Journal of Arthropod-Borne Diseases 6, 1–17. 23293774 PMC3528173

[ref105] Yaghoobi-Ershadi MR, Akhavan AA and Mohebali M (1996) *Meriones libycus* and *Rhombomys opimus* (Rodentia: Gerbillidae) are the main reservoir hosts in a new focus of zoonotic cutaneous leishmaniasis in Iran. Transactions of the Royal Society of Tropical Medicine and Hygiene 90, 503–504. 8944255 10.1016/s0035-9203(96)90295-3

[ref106] Yaghoobi-Ershadi MR, Shahbazi F, Darvishi M, Akhavan AA, Jafari R, Khajeian M, Rassi Y, Soleimani H, Shirzadi MR, Hanafi-Bojd AA, Darabi H, Arandian MH, Sanei-Dehkordi A and Heidari M (2013) Molecular epidemiological study of cutaneous leishmaniasis in the focus of Bushehr city, southwestern Iran. Journal of Arthropod-Borne Diseases 7, 113–121. 24409436 PMC3875877

[ref107] Yakimov WL and Schokhor NI (1914) Réserches sur les maladies tropicales humaines et animales au Turkestan – II. La leishmaniose cutanée spontanée du chien au Turkestan. Bulletin de la Societe de Pathologie Exotique 7, 186–187.

[ref108] Zakharova A, Albanaz ATS, Opperdoes FR, Škodová-Sveráková I, Zagirova D, Saura A, Chmelová L, Gerasimov ES, Leštinová T, Bečvář T, Sádlová J, Volf P, Lukeš J, Horváth A, Butenko A and Yurchenko V (2022) *Leishmania guyanensis* M4147 as a new LRV1-bearing model parasite: phosphatidate phosphatase 2-like protein controls cell cycle progression and intracellular lipid content. PLoS Neglected Tropical Diseases 16, e0010510. 35749562 10.1371/journal.pntd.0010510 PMC9232130

[ref109] Zhang WW, Ramasamy G, McCall LI, Haydock A, Ranasinghe S, Abeygunasekara P, Sirimanna G, Wickremasinghe R, Myler P and Matlashewski G (2014) Genetic analysis of *Leishmania donovani* tropism using a naturally attenuated cutaneous strain. PLoS Pathogens 10, e1004244. 24992200 10.1371/journal.ppat.1004244 PMC4081786

[ref110] Zhang JR, Guo XG, Chen H, Liu JL, Gong X, Chen DL and Chen JP (2019) Pathogenic *Leishmania* spp. detected in lizards from northwest China using molecular methods. BMC Veterinary Research 15, 446. 31818287 10.1186/s12917-019-2174-4 PMC6902407

[ref111] Zijlstra EE (2016) The immunology of post-kala-azar dermal leishmaniasis (PKDL). Parasites & Vectors 9, 464. 27553063 10.1186/s13071-016-1721-0 PMC4995613

